# Recent advances in mapping protein networks regulated by post-translational modifications via proximity tagging

**DOI:** 10.1016/j.cbpa.2026.102683

**Published:** 2026-04-18

**Authors:** Alexander Lin, Mia L. Huang

**Affiliations:** 1 Skaggs Graduate School of Chemical and Biological Sciences, Scripps Research, 10550 N. Torrey Pines Rd., La Jolla, CA 92037, USA; 2 Department of Chemistry, Scripps Research, 10550 N. Torrey Pines Rd., La Jolla, CA 92037, USA

## Abstract

Recent platform innovations are now enabling the resolution of the different ways protein–protein interactions (PPIs) are wired by post-translational modifications (PTMs). Such PTM-aware interactome maps are facilitating the systematic cataloguing of context-dependent network changes. This review highlights developments from 2023 to 2025, focusing on acetylation, methylation, phosphorylation, ubiquitination, redox-regulated cysteine networks, and glycosylation, and outlines analytical and biological frontiers. Together, these advancements are shifting PTM-informed proximal interactome mapping toward mechanism-grounded, actionable frameworks.

## Introduction

Protein—protein interactions (PPIs) are central to cellular biology, orchestrating signaling, molecular trafficking, and the assembly of functional macromolecular complexes—processes that can be dynamically regulated by post-translational modifications (PTMs) [1–4]. These interaction networks create an “interactome,” a context-specific protein neighborhood surrounding a defined “bait” (protein, locus, organelle, or PTM state) at the moment of labeling. Different PTM modifications—set by writers, removed by erasers, and interpreted by readers—add an extra layer of organizational complexity to each network. Acetylation, methylation, phosphorylation, ubiquitination, redox-regulated cysteine networks, and glycosylation shape interactome wiring that varies across subcellular space. Critically, PTMs can be intrinsically dynamic in that installation and removal can occur with distinct kinetics, so the proximal proteins present depend on when and where labeling occurs. Mapping these transient, dynamic, and often sub-stoichiometric events with conventional pulldown methods remains difficult, especially in the nucleus, where nanoscale organization and rapid remodeling compound the challenge.

Rapidly emerging enzymatic and photocatalytic [5] proximity labeling (PL) strategies address these limits by converting local neighborhoods into covalent records retrievable by liquid chromatography—tandem mass spectrometry (LC—MS/MS, Figure 1). When coupled with PTM-perturbing techniques (e.g. small molecules, enzymes), these strategies facilitate PTM-informed interactome maps [6]. In both methods, the enzyme or photocatalyst generates short-lived reactive species that tag nearby proteins (e.g. with biotin), followed by affinity enrichment (e.g. streptavidin) and quantification via MS. A key differentiator between these strategies is the effective labeling radius. Enzymatic PL (e.g. APEX2 [7,8], TurboID [9], Figure 1a) generates reactive intermediates that react locally with nearby proteins (>10 nm), whereas photocatalytic PL produces reactive species that confine tagging to a smaller labeling radius (<10 nm, Figure 1b). Temporally, APEX2 (an engineered peroxidase) enables seconds-scale labeling pulses, TurboID (an engineered biotin ligase) labels proximal proteins over minutes to hours, and photocatalytic PL platforms provide light-gated labeling within seconds. Integration of these strategies with chemical perturbations—ranging from molecular glues, chemical inducers of proximity, to complexoform-restricted covalent ligands [10] allows for capture of specific PTM assembly states and respective interactome changes. Beyond labeling, chemically induced proximity (CIP) is a complementary strategy that utilizes small molecule-based mechanistic perturbation to rewire specific contacts, revealing otherwise cryptic pathways [11]. For example, a CIP system recently uncovered a Piezo-dependent meiotic checkpoint at the oocyte nuclear envelope, illustrating how small molecules can promote proximity to expose transient interactions [12]. In this short review, we focus on recently developed platforms and applications that resolve such PTM-dependent interactomes.

## Chromatin PTM interactome mapping

Chromatin presents a stringent test for PL as its intrinsic PTM states and nanometer-scale organization require precise, time-sensitive labeling. μMap, developed by the MacMillan group (Figure 1b), is a photocatalytic PL platform that creates nanoscale footprints around specified targets by using blue light (450 nm) excitation of a tethered iridium (Ir) photocatalyst to sensitize a cell-permeable diazirine probe via Dexter energy transfer [13,14]. The resulting short-lived carbene reactive intermediate inserts into neighboring proteins within ~4 nm on millisecond time scales, installing proximal covalent tags retrievable by LC-MS/MS. Coupled to in-nucleo protein trans splicing developed by the Muir group [15], μMap can be installed on nuclear targets, such as histone H3.1, to report the respective interactomes tied to specific chromatin states (Figure 2a). MesoMap extends the μMap strategy from single baits to chromatin-wide coverage by selectively distributing μMap photocatalysts across multiple anchor points [16] (Figure 2b). Importantly, MesoMap captures how histone deacetylase (HDAC) inhibition remodels chromatin-proximal networks, linking enzymatic PTM perturbation with spatially organized interactome remodeling.

Acetylation and deacetylation form fleeting contacts that often elude conventional PL. ProLORT, recently introduced by the Zhang group, addresses this by fusing APEX2 to HDAC8 [17] (Figure 2c). With the addition of hydrogen peroxide and biotin-phenol, APEX2 generates biotin-phenoxyl radicals that tag proximal HDAC8 proteins. An orthogonal enrichment “trap” further enhances the selectivity of proximal protein enrichment by incorporating photo-crosslinkable amino acids encoded within HDAC8 to enable covalent tagging of proximal substrates. Importantly, ProLORT uncovered CTTN (cortactin) as a new target, illustrating how brief labeling windows can reveal transient acetylation networks.

Methylation is another key modification that regulates gene expression and genome stability, but mapping proteins at specific methylated loci has been challenging. Qian et al. recently reported the development of SelectID, which combines a catalytically dead Cas9 fused to a biotin ligase (dCas9-TurboID) with a methyl-DNA-binding module (Figure 2d) to label proteins at 5-methylcytosine (5-mC)-decorated genomic sites [18]. With the addition of biotin, TurboID generates biotin-5′-AMP, a reactive intermediate that tags neighboring proteins. Applied to repetitive elements, SelectID identified CHD4 as a potential repressor of transcriptionally active LINE-1 by engaging its 5′ UTR promoter region, demonstrating locus-targeted, PTM-resolved discovery of methylated, chromatin-associated interactomes in live cells.

## Phosphorylation with phosphatase and kinase mapping

Phosphorylation is a dynamic PTM that can rewire PPIs, and identifying the responsible kinase and phosphatase substrates remains an important endeavor. To map phosphatase substrates at near-physiological levels, the Tonks group combined substrate-trapping mutagenesis with biotin ligase proximity labeling [19] (Figure 2e). In this strategy, a BirA biotin ligase produces biotin-5′-AMP from biotin, covalently tagging proximal proteins within ~10 nm. BirA is fused to a catalytically dead, binding-competent mutant protein tyrosine phosphatase (PTP) that retains substrates long enough for covalent tagging and LC—MS/MS identification without forced stabilization, thereby preserving the native enzyme—substrate environment. Applied to HER2-positive and trastuzumab-resistant breast cancer cells, this strategy nominated previously unknown partners in the PTP1B interactome. To expand substrate coverage, PEPSI (PUP-IT Enhanced Protein Tyrosine Phosphatase Substrate Identification) integrates substrate trapping with PUP-IT peptide-transfer labeling [20] (Figure 2f), enabling durable tagging of neighbors around PTPs. Zhang et al. demonstrated the PEPSI approach by profiling SHP1 substrates and identified THEMIS as a previously unrecognized substrate, linking SHP1 proximity to T-cell receptor signaling out-puts [21].

## Ubiquitination with ubiquitin site profiling and DUB proximity mapping

Ubiquitination is a reversible PTM that regulates protein stability, trafficking, and signaling. Ubiquitin (Ub)-enzyme (e.g. E3-ligases) and deubiquitinase (DUB) substrates often form transient, context-specific contacts, making interactome mapping a well-suited technique to capture dynamic Ub-dependent interactomes. Ubiquitin-specific PL concentrates biotinylation on E3 ubiquitin ligase substrate interactions by genetically fusing biotin ligase BirA to an E3 ligase and supplying biotin-acceptor-tagged ubiquitin. With the addition of biotin, BirA drives biotinylation of BAP-tagged ubiquitin and biotinylated ubiquitin is then transferred to proximal E3 substrates (Figure 2g). Three recent implementations—BioE3 [22], E-STUB [23], and Ub-POD [24]—demonstrate that this strategy nominates E3 ligase substrates with high specificity, supporting both targeted protein-degrader applications and generalized substrate mapping.

To resolve deubiquitinase (DUB)-proximal events with site-level detail, the Kessler group developed a proximal-ubiquitomics workflow that couples APEX2 PL to diGly (K-ε-GG) ubiquitination enrichment [25,26]. An APEX2-tagged DUB (Figure 2h) biotinylates neighboring proteins and, after tryptic digestion, K-ε-GG—bearing peptides are captured using anti-K-ε-GG antibodies to identify sites of proximal ubiquitination. Applied to USP30, this strategy detected changes in local ubiquitination and nominated LETM1 as a candidate substrate, linking DUB proximity to functional ubiquitin remodeling.

## Cysteine redox networks and proximity mapping

Cysteine residues undergo reversible covalent modifications that can act as redox switches, rewiring local interaction networks. Recent developments by the Backus group couple TurboID PL with redox-state-informed chemoproteomics to characterize the “cysteineome” (Figure 2i), via Cys-LoC (Cysteine-Local Capture) and Cys-LOx (Cysteine-Local Oxidation) [27]. In Cys-LoC, TurboID biotinylates proximal proteins within a defined subcellular compartment, while cysteines are capped with iodoacetamide alkyne (IAA). Subsequent click chemistry of IAA-tagged peptides to biotin-azide enables capture of site-specific biotinylated cysteine peptides. Cys-LOx similarly utilizes TurboID for spatial anchoring (e.g. within mitochondria) and biotinylation of proximal proteins, but caps reduced cysteines with isotopically labeled light (IPIAA-L) and heavy (IPIAA-H) isopropyl IAA probes, with the ratio of IPIAA-L to IPIAA-H reporting oxidation state at each site. Together, these strategies map cysteine abundance and oxidation with subcellular context and, report the sites of redox changes along with proximal co-localizing partners.

## Glycosylation with protein-anchored mapping and glycoform-dependent readouts

Glycosylation is a crucial PTM that shapes immunity, signaling, trafficking, and protein stability [28], yet its combinatorial complexity and lack of a genomic template render glycosylation-dependent interactome mapping uniquely challenging. Enzymatic PL enables fusions to glycoproteins or glycan-binding modules to tag proximal partners in live cells. Photocatalytic PL provides a complementary, light-gated approach with nanoscale labeling radii for glyco-aware measurements.

We have been using APEX2 to resolve the interactomes of glycan-binding proteins, which have conventionally evaded discovery due to their weak and transient binding characteristics [29]. Recently, we developed an APEX2-FGF2 fusion protein (Figure 3a) to enable mapping of the FGF2 interactome [30], highlighting its glycosaminoglycan-dependent protein partners and illustrating the capability of APEX2-catalyzed proximity labeling to reveal proteoglycan-mediated interactions that evade traditional pulldown methods. Additional heparan sulfate perturbations (e.g. heparin competition or enzymatic digestion) further validated glycosaminoglycan dependence.

Complementary to biotin-transfer PL, proximity oxidative strategies can reveal glycan—protein interactions via localized chemical footprints. Xie et al. introduced protein oxidation of fucose environments (POFE), in which cells are metabolically labeled with a synthetic azido-fucose handle that is conjugated to an FeBABE radical generator. Hydrogen peroxide-mediated generation of hydroxyl radicals oxidizes amino acid residues proximal to the labeled fucose. Using oxidation signatures, fucose-mediated interaction networks for Galectin-3 and Galectin-3 binding protein were recon-structed [31], demonstrating that POFE can identify novel fucose binding partners.

Epidermal growth factor receptor (EGFR) is a heavily N-glycosylated transmembrane glycoprotein whose glycan composition can modulate different biological functions. To resolve the interactome of EGFR, Seath and colleagues developed a red-light PL platform that couples sulfonium diazo probes with a singlet oxygen-generating (SOG) photocatalyst [32] (Figure 3b). In practice, EGFR is targeted with a cetuximab-chlorin e6 SOG photocatalyst (Ce6) conjugate and red light (~660 nm) excitation [33] of Ce6 results in EGFR-proximal protein tagging. A dual carbene strategy using sulfonium diazo probes was implemented in parallel, enabling multi-range labeling of the EGFR interactome. Together, SOG photocatalysis and carbene labeling provide a complementary, glycosylation-informed route to profile glycoprotein interactomes and can expand coverage relative to only carbene-based strategies.

Xu et al. explored the use of a fusion protein joining N-acetylgalactosaminyltransferase-3 (GALNT3) with TurboID (Figure 3c) to map the GALNT3 interactome, revealing proximal O-glycosylated partners [34]. GALNT3 initiates O-linked glycosylation through attachment of N-acetylgalactosamine (GalNAc) to proteins, a process commonly thought to exist primarily in the secretory pathway. Notably, many GALNT3-associated proteins were nuclear-organized, suggesting that O-GalNAc modification or GALNT3-associated complexes may also occur in nuclear compartments.

Glycoengineering remodels the composition and distribution of glycans on a cell or protein surface [35] and has recently emerged as a method to interrogate glycosylation-dependent interactomes. Liu et al. highlight the development of GAP—MS (glycan-dependent affinity purification-mass spectrometry, Figure 3d), in which cells are first subjected to metabolic remodeling of glycan profiles with strategies such as enzymes (e.g. PNGase F to remove N-glycans) or small molecules to generate defined glycan phenotypes [36]. Combined with selective affinity capture, the target glycoprotein and neighboring proteins can be enriched for subsequent LC-MS/MS identification.

Sialylation, characterized by the addition of sialic acids to the terminal glycans of oligosaccharides and glycoproteins, is a post-translational modification with diverse molecular functions. The MacMillan group developed a glyco-targeted adaptation of their μMap technology, referred to as GlycoMap [37] (Figure 3e). GlycoMap selectively profiles sialylated cell-surface glycoproteins. The initial addition of Ac_4_ManNaz facilitates the metabolic incorporation of azidosialic acids (SiaNAz) onto cell surfaces. An iridium photocatalyst is then recruited to sialylated sites via bioorthogonal ligation (e.g. SPAAC). Applied to membrane receptors governing ethanolamine trafficking, this approach highlighted that zinc—receptor interactions are dependent upon sialylation, directly linking a specific glycan feature with changes in local interactomes. To profile sialylation by recognition rather than metabolic editing, Wu et al. leveraged the glycan specificity of siglecs (sialic acid-binding immunoglobulin-like lectins). By fusing the extracellular domain of Siglec-9 to APEX2 and an IgG1 Fc (for avidity, Figure 3f), a chimera was created that proximity labels counterreceptors on native membrane environments [38]. Using this fusion, desmoglein-2 (DSG2) was identified as a Siglec-9 counterreceptor, illustrating how lectin-guided PL can reveal glycosylation-defined interactomes. Taken together, these approaches can map sialylation-aware interactomes across both cells and vesicles.

## Future directions

Recent advancements in PL and covalent chemoproteomics turn PTM states into neighborhood-level maps across chromatin, phosphorylation, ubiquitination, and glycosylation. Together, these approaches capture transient, context-dependent interactions and increasingly connect PTM wiring to functional outcomes. PTMs not only remodel interactome networks but can also reprogram small-molecule engagement, a factor crucially important for therapeutic discovery. In recent collaborative work, Li et al. demonstrated that phosphorylation and N-glycosylation significantly modulate ligand binding for hundreds of different proteins [39]. Notably, KRAS inhibitor activity varied depending on phosphorylation state. In addition, activity-based protein profiling (ABPP)-guided covalent ligands from the Cravatt group [40] can engage reactive, PTM-sensitive residues to acutely perturb complexes. This perturbation can be paired with proximity labeling to convert ABPP-probe target engagement into measurable PTM-dependent interactome changes. These findings argue for incorporating PTM context into target validation, drug design, and therapeutic resistance mapping.

Looking ahead, four trajectories stand out. First, *in vivo* implementations—as demonstrated by Takato et al. using PhoxID to identify the proximal interactome of AMPA receptors (AMPARs) in live mice [41]—can enable *in situ*, cell-type specific maps. A related future trajectory is cross-kingdom expansion of PL: beyond mammalian systems, there is a growing interest in deploying PL in plants. Recent work has outlined plant-specific considerations for construct and experimental design, labeling conditions, and proteomics workflows. Inherent challenges such as endogenous biotin metabolism and unique cellular features (e.g. compartmentation and cell wall) make expanding the interactome of plants particularly difficult [42]. However, continued developments in PL platforms and proteomics can enable more sophisticated approaches that expand upon the dynamics of PL within plants and other non-mammalian systems. A further extension moves beyond protein—protein interactions to protein—nucleic acid interactome networks, where proximity labeling can capture RNA or genomic loci-proximal proteins and, conversely, RNAs neighboring proteins of interest. Nucleic acid modifications are increasingly recognized as biologically important regulatory signals and PL offers a route to characterize modification-associated molecular interactomes. A recent example uses APEX2-based proximity labeling of RNA modification writer METTL1 to map which factors are involved in RNA processing and trafficking [43]. More broadly, emerging modalities such as glycoRNA interactions with RNA binding proteins at the cell surface [44] suggest that glycosylation-modified nucleic acids can form distinct interaction networks that could be characterized using proximity labeling-based concepts. Second, spatial and single-cell resolution by coupling PL to spatial barcodes or imaging-linked readouts can resolve PTM-dependent interactomes in rare cell states or heterogeneous tissues. Third, co-enriching or sequentially profiling different PTMs can multiplex PTM crosstalk. Integrative global multi-PTM studies investigating the concurrent interplay between phosphorylation, acetylation, and thiol oxidation have shown that layered PTM measurements reveal coordinated regulation. Extending this paradigm to the same proximity-labeled sample can help quantify combinatorial PTM-regulation within a single neighborhood. Fourth, computational integration of PL with structure/motif networks will convert proximity hits into predictive, mechanism-anchored interaction maps. Together, these advances will help further move PTM-aware interactome mapping from descriptive atlases to actionable frameworks that connect molecular mechanism with therapeutic opportunity.

## Figures and Tables

**Figure 1:**
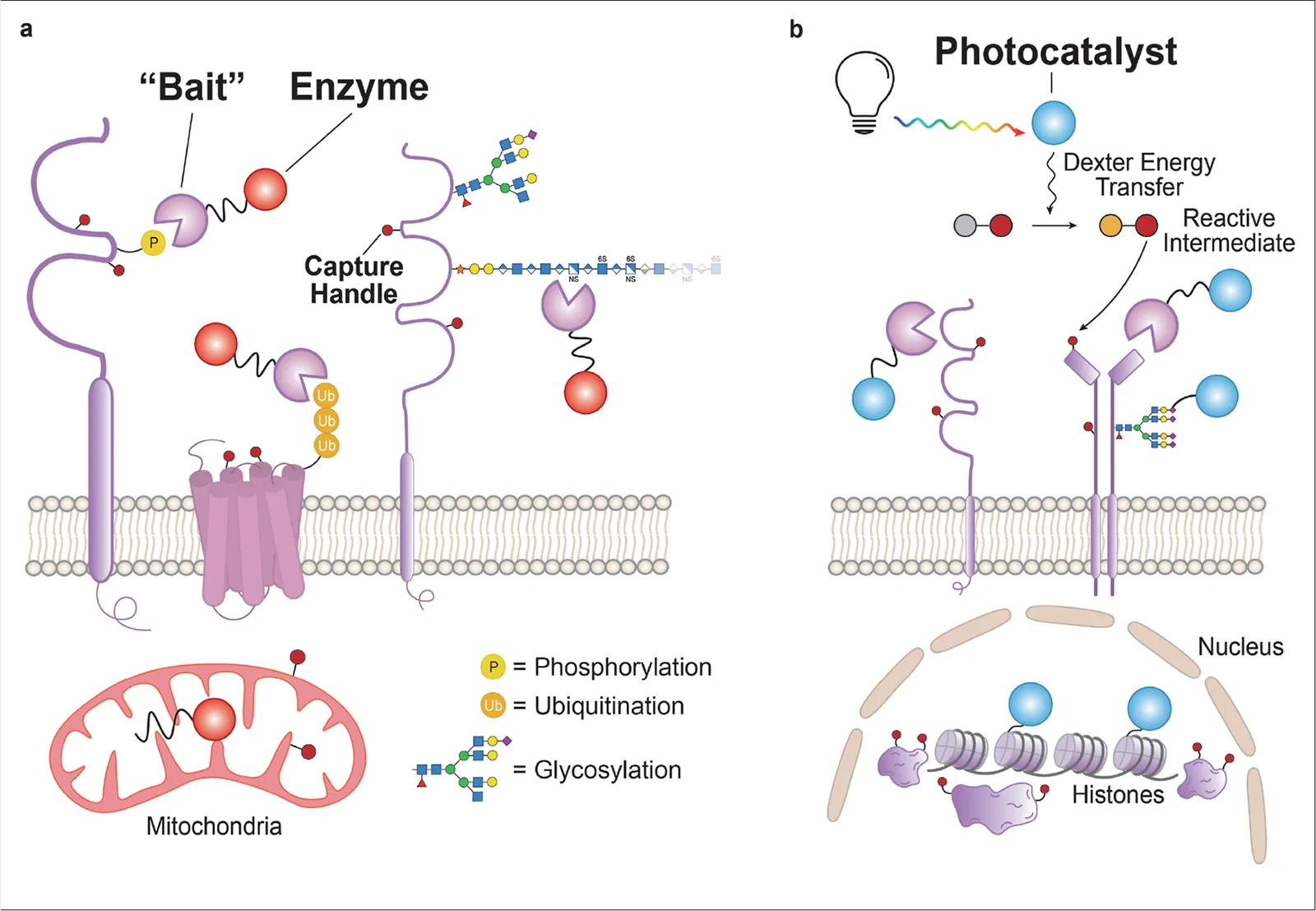
Summary of proximity labeling platforms for PTM-resolved interactome mapping. **(a)** Enzymatic proximity labeling covalently tags neighboring proteins for enrichment and mass spectrometry-based identification. Fusing a catalytic biotinylating enzyme (e.g. APEX2, TurboID) to a “bait” protein of interest–or an intracellular compartment-targeting sequence–enables the tagging of proximal protein interactors. **(b)** Photocatalytic proximity labeling utilizes a light-gated photocatalyst to tag proximal protein interactors. Illumination generates short-lived reactive intermediates that install biotin on neighboring proteins, enabling rapid intracellular or extracellular interactome mapping.

**Figure 2:**
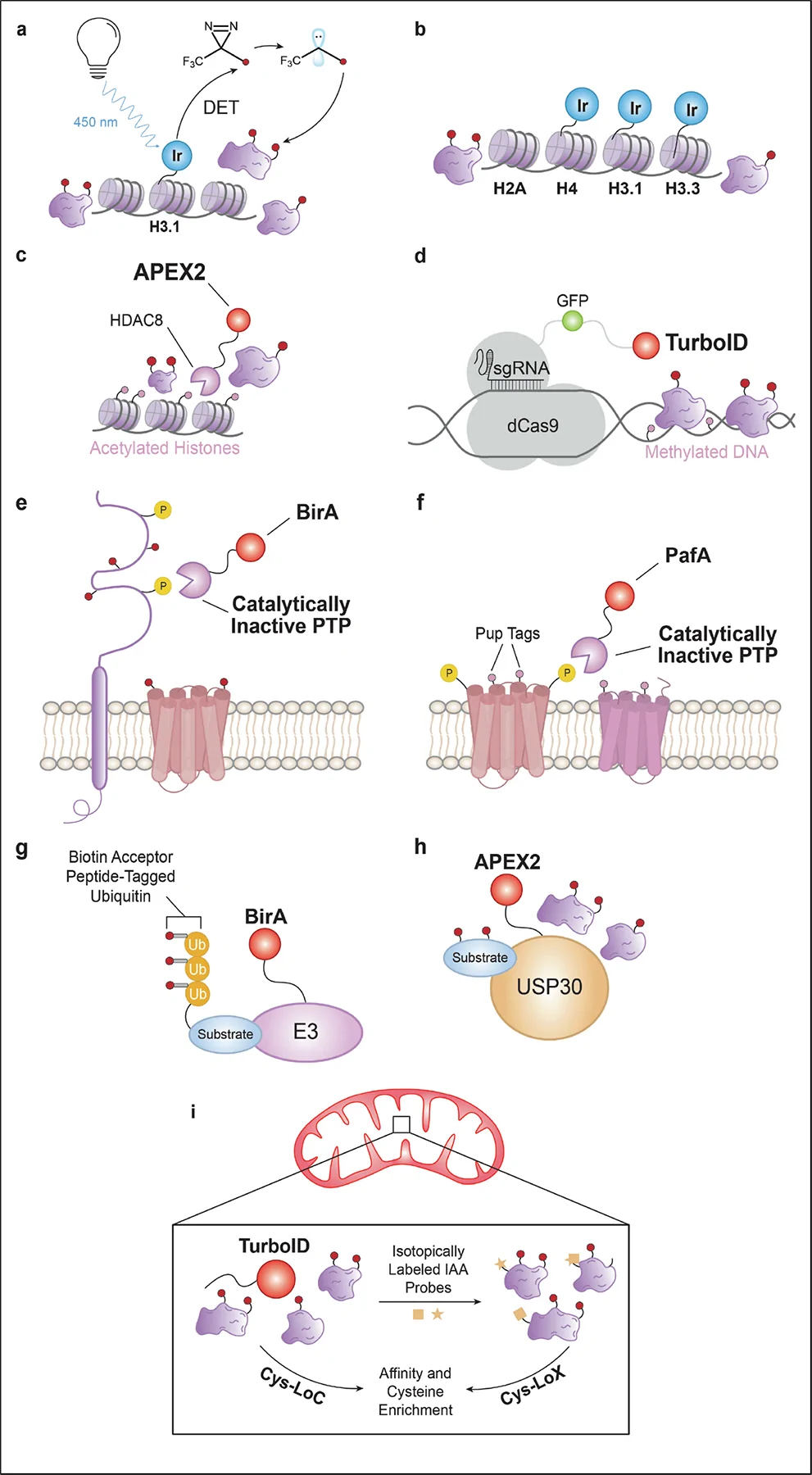
PTM-dependent interactome mapping with enzymatic and photocatalytic proximity labeling strategies. **(a)** μMap installs an iridium (Ir) photocatalyst on histone H3.1. When illuminated by blue light (450 nm), the catalyst transfers energy to a diazirine probe intermediate via Dexter energy transfer(DET) to generate short-lived, reactive carbenes that covalently tag neighboring histone-proximal proteins with enrichment handles. **(b)** MesoMap expands μMap from single histone bait to chromatin-wide coverage by incorporating Ir photocatalysts across multiple different histone anchor points. **(c)** ProLORT utilizes an APEX2 (ascorbate peroxidase 2)-HDAC8 fusion protein in parallel with an orthogonal enrichment trap to interrogate HDAC8-proximal substrates. A brief window of hydrogen peroxide (H_2_O_2_)-catalyzed labeling reveals transient deacetylase networks. **(d)** SelectID combines a catalytically dead Cas9-TurboID fusion with a methyl-DNA-binding module to label proteins at 5-methylcytosine (5-mC)-decorated genomic sites. **(e)** Substrate-trapping mutagenesis paired with BirA-based (biotin ligase) proximity labeling reveals phosphatase substrates. Catalytically-dead, binding competent protein tyrosine phosphatases (PTPs) fused to BirA biotinylates retained substrates. **(f)** PEPSI integrates PTP-substrate trapping with PUP-IT peptide-transfer labeling. A prokaryotic enzyme (PafA) installs Pup peptide tags on proximal proteins. **(g)** Genetic fusion of an E3 ubiquitin ligase to BirA biotin ligase enables ubiquitin-specific proximity labeling to identify E3 substrates. Supplying biotin-acceptor-tagged ubiquitin directs biotin capture handle installation onto proximal substrates. **(h)** diGly (K-ε-GG) ubiquitination enrichment coupled to APEX2 proximity labeling detects changes in local ubiquitination and resolves DUB-proximal interactors. **(i)** Cys-LoC and Cys-LOx couple redox-state-informed chemoproteomics with TurboID proximity labeling to map the “cysteineome”. These strategies quantify cysteine abundance and oxidation, report sites of redox change, and identify proximal co-localizing partners.

**Fig. 3:**
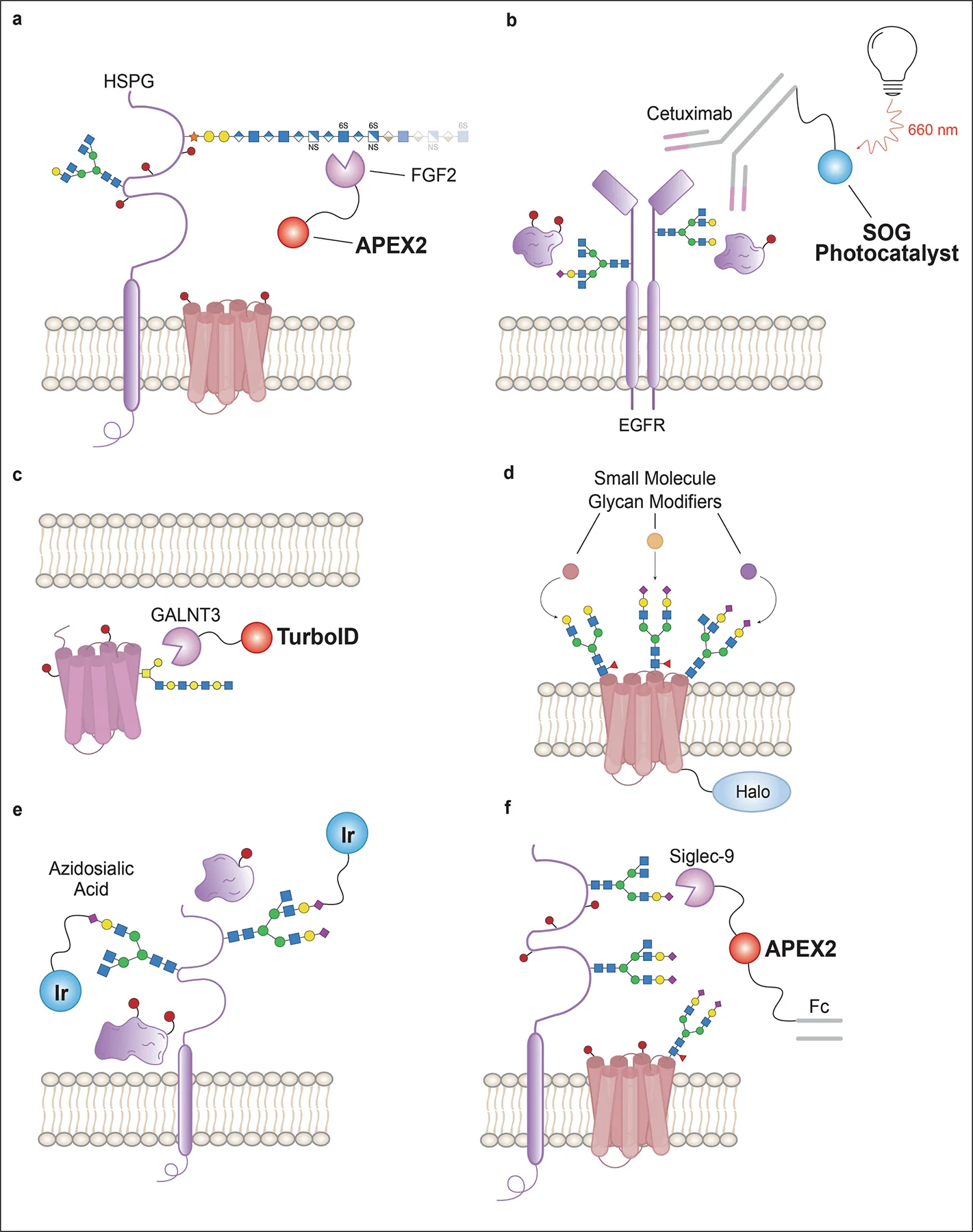
Glycosylation-resolved proximity labeling and interactome mapping. **(a)** An APEX2-FGF2 fusion protein enables FGF2 interactome mapping via peroxidase-catalyzed biotinylation in living cells, revealing glycosaminoglycan-dependent protein partners (e.g. heparan sulfate proteoglycans). **(b)** A red-light proximity labeling system combining singlet oxygen-generating (SOG) photocatalysts (e.g. Chlorin e6) with sulfonium diazo probes enables multi-range labeling of glycoprotein (e.g. EGFR) interactomes. **(c)** GALNT3 initiates O-linked glycosylation by transferring N-acetylgalactosamine (GalNAc) to proteins. A TurboID-GALNT3 fusion characterizes the GALNT3 interactome, revealing proximal O-glycosylated partners. **(d)** GAP–MS (glycan-dependent affinity purification-mass spectrometry) combines small molecule remodeling of glycan profiles with affinity capture (e.g. HaloTag), creating unique glyco-signatures that reveals glycoform-dependent interactors. **(e)** Metabolic installation of azidosialic acids paired with μMap photo-proximity of sialylated sites reveals sialylation-dependent protein interactors. **(f)** A Fc-APEX2-siglec-9 extracellular domain fusion proximally labels siglec-9 counterreceptors on native membrane environments.

## Data Availability

No data was used for the research described in the article.
